# Expectations of Personal Life Development and Decision-Making in People with Moderate Intellectual Disabilities

**DOI:** 10.3390/jintelligence11020024

**Published:** 2023-01-23

**Authors:** José Antonio García-Candel, María Luisa Belmonte, Abraham Bernárdez-Gómez

**Affiliations:** 1 Department of Research Methods and Diagnosis in Education, University of Murcia, 30100 Murcia, Spain; 2 Department of Didactics and School Organization, University of Murcia, 30100 Murcia, Spain

**Keywords:** intellectual disability, expectations, personal development, individual growth, inquiry research

## Abstract

People with intellectual disabilities have a need for personal and social development that is often unknown or poorly understood. The main motivation is the fact that students belong to a group usually excluded from the ordinary educational process. That process is replaced with measures that focus on caring for students rather than promoting their development. The objective of this research is to understand the expectations for personal development and decision-making of students with intellectual disabilities. To achieve the stated objective, a qualitative research based on two complementary approaches, grounded theory and case study, has been employed. The sample (n = 28) was drawn from a specific study program for the training and development of people with intellectual disabilities at the University of Murcia, called “We are all Campus”. We aim to identify the different perceptions of their reality and potential for personal development, to understand their decision-making and what motivates them. Likewise, we investigate the self-perceptions of students with intellectual disabilities have and their understanding of their future life expectations. The main conclusions show that the training program represents an opportunity for students’ progression and personal development. Thus far, the expectations of the students have remained the same, focusing on their work and social inclusion.

## 1. Introduction

This research aims to understand the expectations for personal development as well as the decision-making of students with intellectual disabilities (ID). It has been developed in a university program called “We are all Campus”. The various goals set internationally, such as the SDGs, are included in the purpose of this program, the focus of which is the inclusion of people with intellectual disabilities, because the Spanish educational reality is far from being inclusive for certain groups yet, especially people with ID (Alcaraz and Arnáiz 2020).

Today’s society is characterized by a desire to improve people’s quality of life. One way to achieve this is through the protection of citizens’ rights. Nevertheless, minority populations have been excluded from current ideals of equality (Víquez et al. 2019), such as people with Intellectual Disability (ID). According to the American Psychiatric Association (2013, p. 17), intellectual disability “is a disorder that begins during the developmental period and includes limitations in intellectual functioning as well as in adaptive behavior in the conceptual, social and practical domains”. This means that this group has usually had to fight against many obstacles when it comes to being the protagonists of their own life stories. Consequently, their inclusion in society has also been hampered. For this reason, the present study aims to learn more about the perceptions of people with ID, including the topics of their personal and social development, their expectations and decision-making.

Some time ago, we embarked on a transition from a view of disability that was focused on a pathological perception, to another that takes into account the support needed by people with ID (Heras et al. 2021. There is no doubt that, over the years, a transformation has been reflected in terms of the conception of disability (Víquez et al. 2019) and that these attempts at ideological change seek to set aside static scenarios, focusing on the construction of a new reality for this group in society. This transition has enabled a movement towards the social model of disability, built as a result of the various social, political and educational changes. These have focused on interventions with people, their needs and potentialities, their struggle for independence, their possible labor productivity and, after all, their integration into society (Shakespeare 2010).

In short, today intellectual disability (ID) is seen from different complementary angles: the biomedical perspective (focused on the genetic and psychological elements that originate said ID); the psychoeducational perspective (focused on the intellectual, psychological, behavioral and learning); the sociocultural perspective (which analyzes the interaction of people with their environments); and the perspective of justice (where all people have the same human and legal rights) (Schalock 2018).

For all these reasons, it is reductionist and unfeasible to simply annul the will of citizens with some degree of intellectual, cognitive or psychosocial disability, since this would imply taking away their recognition as subjects of law under equal conditions, seriously damaging their dignity and autonomy. The social model aims to provide tools to ensure the development of people with disabilities in different contexts, making efforts to “balance access to the full exercise of their rights and opportunities in a society within which they can freely and with dignity develop their own life plans and projects” (Victoria 2013, p. 1094). Thus, these groups can become active participants in the socio-community sphere (Víquez et al. 2019). That is, this transition in the models of intervention with people with ID has facilitated a greater openness towards the social world, an emergence that has generated feelings of freedom and well-being (Mañas et al. 2020).

However, people with ID have to deal with the beliefs of family members, professionals, society in general (and even themselves) regarding their own transition to adult life, especially at the work level, on a daily basis. Low expectations inevitably hamper their chances of development (Lent and Hackett 1994) and can lead to poor functional competence in terms of job skills (O’Brien et al. 2001; Fitzgerald and Betz 1994). It becomes crucial to involve the person with ID in decision-making in the various social contexts in which they participate, especially in the work environment, due to the repercussions that this has on the individual’s own life (Moreno and Belmonte 2022).

### 1.1. The Training Program We Are All Campus

The training program “We are all Campus” (Belmonte and Bernárdez-Gómez 2021; Moreno and Belmonte 2022) of the University of Murcia (Spain), framed at the national level within the UniDiversity project, considers that differences must be incorporated into the classrooms and, by extension, into daily and community life.

The University Degree in Own Studies life incorporated into the classrooms and due to the repercussions that this has on the individual, the call for grants from the National Organization of the Spanish Blind Foundation (ONCE), allows young people with intellectual disabilities to study at the University of Murcia in preparation for getting a job. Actually, this is a training program for the improvement of autonomy and socio-inclusion work, as well as developing motivation for learning and the responsible performance of tasks. For the University of Murcia, it means establishing an inclusive training and normalization system in the environment of the university community with the corresponding benefits that this entails, such as social, affective and personal balance, related to the feeling of non-exclusion (Mañas et al. 2020); academics or social justice education; and finally, labor or employability, concerning the number, type and permanence in employment achieved by the person with disabilities (Gavín and Molero 2020).

Specifically, participation in this program is open to people between 18 and 30 years old who have an officially recognized intellectual disability equal to or greater than 33%^1^; additionally, they must be registered in the National Youth Guarantee System, have the ability to move independently, and possess basic academic skills of minimum competence in reading, writing and calculation, among others.

It should be noted that the training contents are organized into two academic courses within the program: the first course provides an introduction to the work environment, while the second course provides more specialized knowledge about employment. Both courses have been organized in blocks: Inclusion and Social Participation; Personal Development and Autonomy; Job Skills; Vocational Training; Supported Employment Practices. In other words, it is a project aimed at the comprehensive training and job preparation of people with ID, organized into different functional, humanistic and professional subjects and taught by university teaching staff. Furthermore, complementary extracurricular activities are included, some of which are shared with university students from other official degrees. The program also offers the opportunity to undertake an internship in various entities and external companies in the area, always with the support of job coaches, with the aim of obtaining the most realistic work experience possible.

Hence, this program, which directly aims to improve the personal skills of people with ID for a successful transition to active adult life, strongly advocates the participation of this group as full members in its community. This necessarily implies promoting their potential; that is, providing resources to increase the labor and social inclusion of this group. In Spain specifically, this group finds it very difficult to continue with their academic or professional training beyond Compulsory Secondary Education.

### 1.2. Objectives

The general objective proposed for this research is to know the expectations for personal development and decision-making of students with ID in the program “We are all campus”.

Relying on this, we formulated the following specific objectives:

1. Fathom the main themes around which the expectations and decision-making of students with ID are circumscribed.
2. Point out possible differences between students in the initial and advanced courses and between different genders.
3. Characterize the type of sentiment produced by each of the emerging themes.
4. Establish possible relationships between each of the categories that emerged in the expectations and decision-making of students with ID.

## 2. Materials and Methods

### 2.1. Design

The design that was selected in order to achieve the proposed objectives was of a qualitative nature (Taylor et al. 2015). Two different qualitative methods have been used in a complementary way to develop the research; on the one hand, the grounded theory and, on the other, the case study. That is, different methodological designs have been followed to adapt the particularities of the research to the context in which it is developed. It is a grounded theory study of the development of people with ID, focused on the description of a moment in the lives of the subjects, the path followed to reach the present moment and its future projection in its natural context (Hernández-Sampieri et al. 2018). The central idea of this type of research is that it seeks to understand a situation from a holistic perspective by observing an object of study from different perspectives (Bernárdez-Gómez 2022). The researcher evaluated different beliefs of the subjects and made the necessary inferences to understand the motivations of the people in front of him. In this way, within grounded theory, we do not act with a pre-established framework in the investigation, but rather it emerges from the data, giving more value to the results and their discussion. Grounded theory gives flexibility to the researcher to adapt to the situations that arise during the investigation.

In addition, this research follows a case study desig, since it is governed by the premise of responding to research questions and dynamics present in a specific context (Taylor et al. 2015). Thus, the case study framed in this work is marked by its development in the University Degree of Own Studies^2^ offered by the University of Murcia.

### 2.2. Sample

The sample size for the case study far exceeds that indicated by Hernández-Sampieri et al. (2018), who noted that a total of 12 would be sufficient. It should be added that to validate the sample of this research it has been observed that there was a sufficient theoretical saturation in the analysis of the data. Thus, the participants were the 28 subjects enrolled in the academic year 2021/2022. As mentioned in the introduction, this program is designed for people with a recognized ID greater than 33%. These young people must possess autonomous mobility skills and basic skills in numeracy and reading and, in addition, they must be between 18 and 30 years old and have been enrolled at some point in other social insertion programs. The sample is distributed in the two courses of the program, with a total of 6 women and 9 men in the first year, and 9 women and 4 men in the second year.

### 2.3. Data Collection

Due to the difficulties that may result from undertaking research work with people with ID, data collection has been carried out using two complementary and integrated techniques (Onghena et al. 2019), namely a semi-structured interview and a biogram. Through the general questions posed in the interview, the subjects have given various perceptions of their reality and potential personal development. The interview guide has been validated by expert judgment. The semi-structured interview was developed based on a series of key or general questions for each of the topics of interest (for example, what expectations did you have when you were younger? Have they been fulfilled? What decisions have you made to fulfill them?). In the case of needing to explore some of the topics in greater depth, there were some deepening questions (probes) on specific aspects. Some of the deepening questions were: What do you usually choose throughout the day? Would you like someone to choose for you? Why is it important to choose things? To make the questions more accessible, they were supplemented with examples of answers that did not condition the subject.

**Figure 1:**
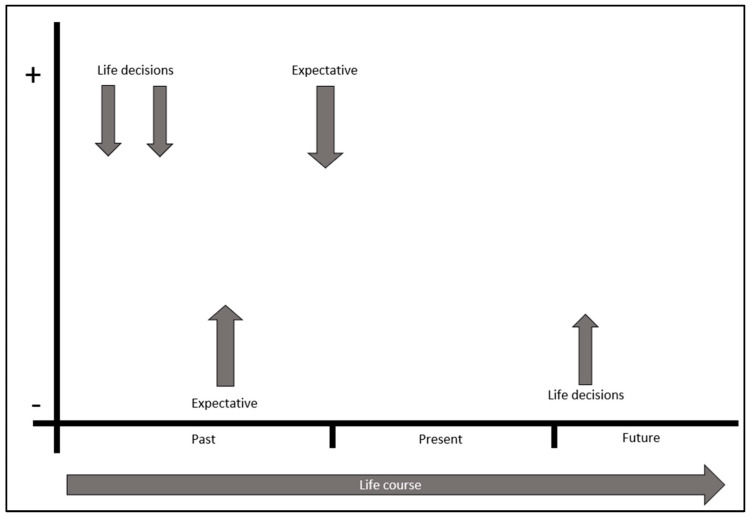
Biogram template used to point out the different decisions and expectations throughout respondents’ life paths.

To help identify the different perceptions of the subjects, the biogram was applied simultaneously to the interview. This is a basic biogram of their life trajectory (Figure 1), in which their past, present and future is reflected. It is intended to indicate the moments at which they have had to make decisions and what has motivated them. Moreover, the application of this biogram served as a visual tool in enabling the interviewees. Similarly, they were questioned about their general state and perception of themselves at the present time, as well as their potential, and then they were asked about their future expectations and the decisions they must make in achieving them. Respondents were asked about their perception (positive or negative) about each of the decisions made and their expectations. In this way, the influence of sentiment in the data analysis could be assessed. Each of the subjects wrote the different decisions and expectations throughout their life trajectory on the biogram timeline.

### 2.4. Data Analysis

Data analysis has been developed using the technique of grounded theory (Alarcón et al. 2017; Franco and Morillo 2016). This method of analysis, together with the support of the ATLAS.ti V22 analysis software, is the optimal way to delve into the data and make it emerge meaningfully to the researcher (Patton 2002). This is intended to reconstruct the content provided by the interviewees, transforming the data to raise valid interpretations. In this way, the procedure established by Strauss and Corbin (2002) has been followed, whereby the information is worked inductively and interpretatively so that data from the categories of the analysis emerge. The procedure, although developed in the stages proposed below, is a recursive process in that it returns to previous phases as many times as necessary to reach the correct interpretation:

1. Open coding, at the initial moment of the analysis, identifies different superficial concepts, categorizing data into manageable units that help in the generation of categories.
2. Axial coding, at the first moment of theoretical construction. The central categories by which we will structure our discourse emerge. The core categories arise from the most relevant codes. In this phase, we work with the citations linked to the codes to begin to generate content and the different relationships that may arise between them. Here, the ATLAS.ti V22 Analysis tool of co-occurrence was especially relevant.
3. Selective coding, last proposal by Strauss and Corbin (2002). The purpose of this is to establish relationships through which the knowledge of the studied phenomenon can be deepened.

Finally, in the coding, eight central categories emerged that will be developed below, in addition to the four sociodemographic categories and the three of the sentiment analysis carried out by the program autonomously. The main coder, one of the authors, is a certified ATLAS.ti trainer, which is an added value to the coding and to the coding guide of the collaborators. Additionally, for greater encoding reliability, software intercoder agreement has been practiced. After carrying out this double coding, the coefficient reached in the set of dimensions was 0.81 (Krippendorff’s cu-Alpha).

### 2.5. Ethics

The project to which this research pertains has been reviewed and approved by the Research Ethics Committee of the University of Murcia (Approval Identification Code: 3408/2021). In addition, all members of the sample participated on a voluntary basis. After the development of the research and the anonymity and confidentiality of their participation was explained to them, the respondents completed a form in which they gave their informed consent to participate.

## 3. Results

### 3.1. What Are the Main Issues You Are Discussing?

The first result that emerges after performing an inductive coding is the categories or topics that the students talk about. Throughout the discussions of various expectations and decisions that had been made during their lives, the students frequently mentioned a series of issues related, fundamentally, to their personal development in society. Thus, the categories/topics listed below emerged:

- Personal expectations: expectations of a personal nature through which they seek their own development, autonomy or ability to choose. “May all my dreams come true”. (D1:60)^3^. This category was used when participants talked about something specific they wanted to happen. Many of the items refer to goals that the respondents intend to achieve. These expectations are tangible and real for them, although they do not have a very clear path towards achieving them.
- Social relationships: mentions of the different social relationships they can establish and the forms they take. “I am proud to have these colleagues because they are very good people and also to have scored well in the exams”. (D1:67). These types of relationships are not as close as personal ones can be. However, they have a strong effect on these people because, in many cases, this is their first contact with society and their first stage of social development. In addition to classmates, they also manifest relationships of this type with co-workers or other people they meet in their day-to-day lives.
- Interpersonal characteristics: each respondent’s intrinsic characteristics and how these affect their development or occur in the different facets of their lives. “To have learned with my failures and to mature as a person”. (D1:99). Interpersonal characteristics may refer to two specific issues: specific abilities that are, or are not, in them, and over which they may assume to have a certain control, such as their ability in mathematics; and characteristics derived from aspects that they do not believe they can control or that are difficult to modify or improve, such as those related to their disability.
- Training: references to training they have acquired or training they want to undertake. “I choose to learn new things like cooking recipes, sports, languages”. (D1:79). The participants understand that there is a need to train constantly, and they express it this way. Training is the means by which they can improve and acquire the skills or abilities that are necessary for their development or to develop leisure activities.
- Entertainment and leisure: category used when mentioning leisure activities. “Going for a walk with my friends and monitors”. (D1:113). Closely linked to the other categories, the participants constantly mention the leisure activities that they carry out or those that they perceive as a need. These are the main activities in their daily lives that give them satisfaction.
- Interpersonal relationships: comments concerning the closest relationships, those of family or intimate relatives. “I am proud to have had a family that loves me”. (D1:157). The respondents refer to these when they talk about their closest relationships. Such relationships are especially relevant because they strongly influence daily life and pertain to one’s immediate environment.
- Job expectations: related to the interviewees’ job expectations. “To be able to be autonomous and be able to work tomorrow”. (D1:224). One of the main aspirations presented by students is the desire to get a job which will give them autonomy. This is accompanied by a need to feel useful and valued by society and by the people closest to them. For these reasons, the respondents frequently expressed their expectations of having a job.

If we begin by breaking down the results of the concurrences between the thematic categories and the sociodemographic data, gender and course, it can be observed in Figure 1 that there are significant differences between the genders in two specific issues. Men value interpersonal relationships more (0.29)^4^ and women make greater mention of personal expectations (0.34); that is, their development. With a lower (but also relevant and egalitarian) concurrence between the two genders, we have social relations (co-occurrence of 0.18 in both genders) and the training received or wanted.

### 3.2. Main Differences between Gender and Course

**Figure 2:**
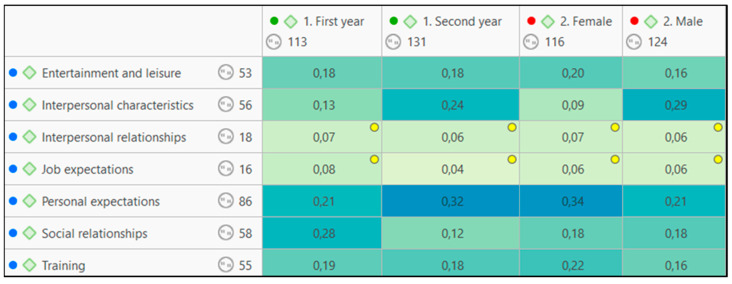
Table of co-occurrences between thematic codes that emerged in the analysis and codes assigned for gender and course on which the student is enrolled^5^.

In terms of a comparison between the students of various courses, it can be observed that, in the first year, the respondents take more account of personal relationships (0.28) whereas, in the second year, students tend to focus more on their personal development (0.32). It is relevant to point out that, in the second year, personal relationships decrease drastically in their concurrence, becoming important to aspects such as interpersonal characteristics (0.24). In both cases, the respondents similarly value topics such as training and leisure, taking into account that both codes are understood to be complementary to other aspects, as can be seen below (Figure 2).

**Figure 3:**
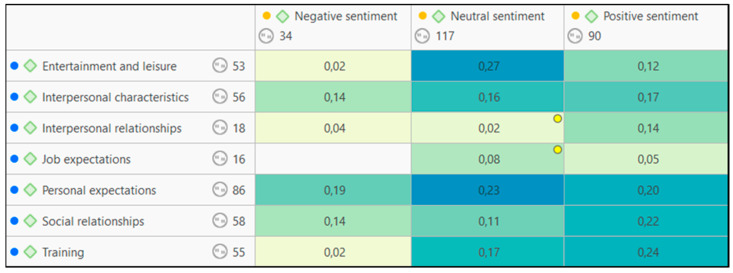
Table of co-occurrence between thematic codes that emerged in the analysis and codes of recognition of feeling.

In addition to the sociodemographic data, it was also relevant to know how the interviewees felt about each of the issues they mentioned; consequently, through the sentiment analysis tool of the ATLAS.ti software, this assessment was made. This tool of the program analyzes the feeling in each of the statements, with the options ranging from positive to neutral or negative feeling. In this way, it is possible to analyze the disposition of the respondents towards each of the emerged categories. Figure 3 highlights the general neutrality that has emerged from the coding. However, it should be noted that the aspect that generates the greatest negative feeling is the respondents’ own personal expectations (0.19). Similarly, training and social relations are particularly relevant issues for them, with coefficients of 0.24 and 0.22, respectively.

Deepening the Research, Feelings and Relationships between the Themes

**Figure 4:**
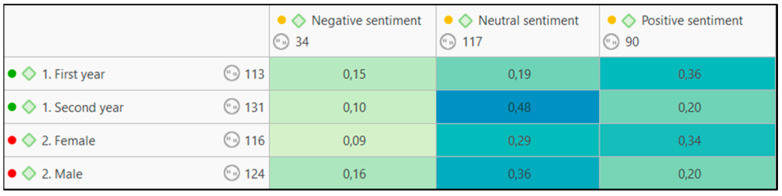
Table of co-occurrence between sentiment analysis codes and sociodemographic codes.

In this regard, if we analyze the feelings or dispositions shown across each of the groups and genders (Figure 4), it will be observed that none of them have a negative disposition. In the case of women, they present a higher prevalence of expectations and decisions of a positive nature (0.34) and the first year group presents the same disposition (0.36).

**Figure 5:**
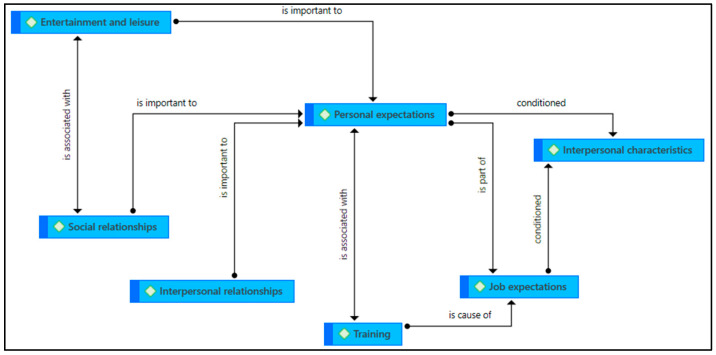
Semantic network of relationships between topics.

Focusing more closely on the themes that emerged in the analysis of data (Table 1), we see that there are aspects with a consistent relationship. These relationships are explained in the semantic network, as shown in Figure 5. Similarly, we are able to observe the coefficient of co-occurrence of each of the codes in the analysis that justifies the relationship shown in the semantic network.

As mentioned, the results show that the main focus of interest among the interviewees is their personal expectations; that is, their individual development and how they can manage it or actions they can take towards it. For this reason, issues such as their relationships, both interpersonal and social—which, in turn, are often associated with leisure and entertainment activities—are important. On the other hand, personal expectations are also strongly linked to training and job expectations, since the participants understand training as a facilitating element of a job. However, all of this will be conditioned by the specific issue of their interpersonal characteristics, which often sow the germ of frustration in their daily lives, as well as rejection by others.

Finally, we must take into account the different stages in the life trajectory of students. Throughout the interviews and the analysis of the data, no notable data or quotes have emerged that indicate a change in the expectations of the students. Therefore, no reference is made to this aspect in the presentation of these results. Likewise, this indicates that they do not present a differentiated perception or evolution in their expectations depending on the stage. This demonstrates that the categories that emerged from the analysis are issues that have been maintained over the years and have not been resolved.

## 4. Discussion

The various findings of this study have revealed the relevant elements in the life trajectory of the subjects studied. No clear evolution has emerged to allow us to define the progress of past, present and future expectations. Despite this, it has nevertheless been possible to illuminate a series of elements of great relevance to the students with ID, as evidenced throughout different points of the discussion.

### 4.1. The Hope Placed in Your Future, on Personal Development

In terms of the specific content of their dialogue, to a greater extent, the interviewees with ID talked about their individual personal expectations. They wanted to develop personally with the ability to make choices, experience autonomy or achieve their own goals. Research has repeatedly shown that having opportunities to participate, learn and get involved in activities—in short, having opportunities for personal development—contributes to an improvement in the quality of life of people with ID (Gómez et al. 2022).

The respondents spoke of their capacity for choice, of autonomy, of their individual potential, but socially and historically, they have been a limited collective that has depended on other people. Now, they want to make their own decisions on basic aspects of their personal development, despite the fact that people with ID experience numerous barriers such as, for example, emancipating themselves (Pallisera et al. 2018).

This personal development becomes vitally important from a perspective of life satisfaction (Ginevra et al. 2018), linked to subjective well-being and a predisposition to positive feelings over negative ones. This is fundamental in personal aspirations in terms of success or failure for the achievement of greater life satisfaction (Gavín et al. 2022): “May my happiness depend on me” (D1:58).

After all, the personal well-being and life circumstances of this group will determine their quality of life (Gómez et al. 2022; Rodríguez et al. 2017), along with their health, psychological well-being, social relationships and environment, where each person develops from an individual perspective regarding these vital values (Kurtek 2020): “May all my dreams come true” (D1:60).

Over the years, the belief that people with ID, due to their sometimes limiting condition and their abilities, are not competent to cognitively value their environment and what happens within it has been perpetuated (Medina and Gil-Ibáñez 2017), sometimes due to aspects related to the specific disability and sometimes due to a difficulty in recognizing and expressing emotions (Scott and Havercamp 2014). This has given precedence to the opinion of third parties over that of the person in question (Gavín and Molero 2020): “I choose my belief, how I dress, who I join and what I want to study” (D1:77).

Thus, people who manage to understand themselves can better recover an optimal state of well-being (Cazalla and Molero 2018; Suriá 2017). Those who can maintain control of their emotional state, whether negative or positive, will present a higher level of satisfaction with life (Cejudo et al. 2016): “Having learned [from] my failures and maturing as a person” (D1:99).

It is very common for this group to encounter difficulties in actualizing their desires, given their sometimes absolute dependence on others to interpret them (Hogg et al. 2001). Fortunately, the planning of services for people with ID is increasing, although it remains a goal to be achieved, since it directly improves the quality of life of people with ID (Gómez et al. 2022; Heras et al. 2021). In addition, it is one of the most relevant variables to analyze the effectiveness of interventions in this group (Gavín et al. 2022): “I am happy with the decisions I choose when I am achieving my goals” (D1:125).

### 4.2. The Importance of Personal Relationships

To a lesser extent, the respondents also value the different relationships they establish, which are often conditioned by their personal characteristics.

Thus, people with ID generally take into account interpersonal relationships, valuing family, their close circle of friends and the sentimental relationships they establish or desire. This is an aspiration, which is sometimes unattainable, since in many cases they do not receive the necessary support to participate significantly in activities that interest them (Mirenda 2014), having more limited social networks than other people, with less frequency of mutually satisfactory interpersonal relationships (Gavín et al. 2022; Hostyn and Maes 2009). So much so, that the absence of meaningful relationships can, in addition to hindering inclusion (Belmonte et al. 2021), condition the leisure of people with ID (Scott and Havercamp 2014). “Going out on Fridays with my partner and Saturday with friends” (D1:3).

Therefore, people with ID are more likely to remain in segregated environments (Heras et al. 2021), encountering greater difficulties in exercising their right to self-determination. Despite this, studies have shown that the experience of living with a disability, far from intimidating an individual, seems to activate a process of struggle and overcoming this problem to successfully face the obstacles that are entailed (Mirete et al. 2022). This does not mean that people with ID do not experience negative moments or emotions, but that positive emotions coexist with negative ones during these adverse circumstances (Folkman and Moskovitz 2000) and these are an aid to the empowerment of the coping capacity to adapt adequately to these circumstances (Shakespeare 2010). “I am proud to make more friends” (D1:25). “I am proud I’ve decided to have a group of real friends on Whatsapp” (D1:121). “Going out with friends for a walk” (D1:49).

Participation or even simply residence in community settings has long been linked to significant improvements in adaptive skills (Conneally et al. 1992; Kozma et al. 2009; Lakin et al. 2011; O’Brien et al. 2001); interpersonal relationships (Emerson et al. 2000; Kozma et al. 2009); increased participation in activities that are revealing to the individual (Conneally et al. 1992; Emerson et al. 2000; McConkey et al. 2016); a more varied lifestyle (Conneally et al. 1992); a less sedentary lifestyle (Kozma et al. 2009); and, after all, greater opportunities for self-determination and decision-making (Emerson et al. 2000; Kozma et al. 2009). “I am happy to have had a big event I went to and also to communicate with people and other colleagues from other parts of Murcia” (D1:65).

### 4.3. Training and Work as a Way Forward

In terms of their personal development, or expectations related to what they can do and how they want to live their life, the respondents felt that they must have autonomy to be able to choose and attend to their own needs as they perceive them. Similarly, they can identify the capabilities that they want and need to develop; even if they are wrong, they will be able to resolve it. This is the case with training and work; it represents an opportunity for them to grow personally and this is what they crave so much.

The main problem is that, from an early age, they have perceived that educational institutions have presented an environment of segregation and exclusion (Heras et al. 2021; Mañas et al. 2020). A person with ID is less likely to engage in an education that takes place within the ordinary system (Emerson et al. 2000; Navas et al. 2017). Moreover, harassment usually constitutes the overriding tone of this setting, becoming too repetitive a reality for people with ID in educational centers. Such criticisms and negative views of people with ID can, over time, erode identity. This identity, despite being moldable and variable, configures the sense, belonging and recognition of people (Gómez et al. 2022) differentiated and excluded, towards them. “I am proud to learn English, which I did not know anything before in my old school” (D1:33).

However, studies have shown an association between people with ID and high levels of empowerment. Feelings of control over their lives, a high level of self-esteem and relatively indifferent reactions to stigma have been observed (Mirete et al. 2022).

That is where the importance of addressing the empowerment of people with ID lies, since it can help to understand the problems of this group, not only from the limitations derived from the disability but also from overcoming these (Suriá 2017). In addition, it is also especially relevant that society assumes an inclusive culture, given that the promotion of diversity in the educational context facilitates the integration of these people with ID (Belmonte et al. 2022). “I am very proud of myself because every day I get up to go to college and to learn new things” (D1:194).

Unfortunately, although there are many actions focused on promoting the self-determination of people with ID, there are still significant barriers to this (Vicente et al. 2018). This is because such a group is particularly vulnerable in terms of the defense and guarantee of their rights (Navas et al. 2017). In addition, the degree of inclusion influences the personal results that this group can obtain in many dimensions, such as their personal development, emotional well-being, their rights, etc. (Hall 2010). For this reason, a real and community coexistence is especially relevant, since “the attitudes of acceptance of difference increase with the knowledge and understanding of the meaning of disability” (Belmonte et al. 2022, p.168) and, after all, this has an impact on their lives (Gavín and Molero 2020). “I want to work in a nursery because I like children and I want to learn how to change a diaper” (D1:184).

Furthermore, it is remarkable that personal expectations are the category in which the greatest negative feeling is shown. However, this is in line with the concept of low expectations being the starting point of a vicious circle that considerably limits the opportunities of people with intellectual disabilities (O’Brien et al. 2001).

Similarly, personal expectations represent the emerging category that attracts the most attention among students with intellectual disabilities in relation to expectations and decision-making. These personal expectations are closely related to personal autonomy and, in this sense, this group can be made to believe that their abilities (in our case, in the work environment) are lower than they actually are (Shogren et al. 2015).

The processes of exclusion, supported by the notion of disability as a factor of inferiority (González et al. 2017; Mañas et al. 2020), keep this group silent. Based on the principle of integration towards the normalization of the lives of students with special educational needs (Conneally et al. 1992), the “We are all Campus” program reflects a firm commitment to the inclusion of people with intellectual disabilities in the university, seeking to turn the voices of silence into voices for change (Belmonte et al. 2021). To this end, it outlines the realization of a training project, capable of responding to individual needs and inclusion, so that young people with intellectual disabilities can participate as full members in their educational community.

## 5. Conclusions

The objective set for the following research was to understand the expectations for personal development and decision-making of the users of the training action “We are all Campus”. Throughout the journey shaped by their needs, opportunities for development, decisions and future perspectives (Shogren et al. 2015), the students of the program have expressed a common concern, which is the opportunities provided extrinsically. Students have voiced their need to choose for themselves, with autonomy and independence. Faced with an education based on care, they have repeatedly requested the acquisition of skills that will help them to function in society.

It should be noted firstly that the categories obtained after coding together provide a panoramic view of the appropriation and internalization processes of the participants in relation to expectations and decision-making regarding their personal development in society. In other words, it provides a group of themes that summarize the conflicts and tensions that are inseparable from the social spaces in which people with intellectual disabilities live.

On the other hand, if we focus on gender, significant differences appear, as men place greater importance on interpersonal relationships (friendships, family, etc.), while women focus on personal expectations (referring to their own development, autonomy or ability to choose, assuming the consequences thereof). Furthermore, we verified that personal and social relationships form the most relevant topic for first-year students; this is in contrast to second-year students, who place greater emphasis on their personal development (process of improvement and growth to achieve dreams and aspirations that contribute to a better quality of life).

It is, therefore, essential socialization of the group of people with intellectual disabilities in these stages, being really beneficial for both people with disabilities and for those who do not have any disability. These values promotes this program TSC, as the interrelationship and coexistence among peers of the same age, because to achieve full inclusion, one must first facilitate the approach of both contexts, promoting a harmonious coexistence.

For this reason, promoting training actions of this type is particularly relevant, not only because they are very scarce, but also because they are enriching and provide a field of action for the implementation of this inclusive reality, which generates active citizens. With this empowerment of people with intellectual disabilities, a greater empowerment of their expectations and self-knowledge is achieved, which has a direct impact on the perception of themselves and the recognition of their capabilities.

### Limitations

To conclude this paper, it is worth highlighting a series of limitations observed in the research and the directions we believe should be followed in this field.

On the one hand, we can point out the following limitations. Methodologically, it has been costly to develop and plan a research study with people with intellectual disabilities. The design required much more effort to adapt to the subjects and to make them understand the process we were trying to follow. We had to point out the difficulties in getting them to situate themselves temporally within their trajectory. Therefore, it was not possible to identify future, present or past expectations.

On the other hand, although the sample is sufficient for a qualitative methodological design, it would be desirable to be able to continue with data collection in other similar projects. This raises the need to carry out research that will enhance and improve the autonomy of students with intellectual disabilities and enable them to become active citizens.

## Figures and Tables

**Table 1:** Table of co-occurrence between emerging categories.

	Entertainment and Leisure	Interpersonal Characteristics	Interpersonal Relationships	Job Expectations	Personal Expectations	Social Relationships	Training
Entertainment and leisure							
Interpersonal characteristics	0.04						
Interpersonal relationships	0.04	0.00					
Job expectations	0.00	0.01	0.00				
Personal expectations	0.05	0.11	0.05	0.05			
Social relationships	0.16	0.07	0.04	0.00	0.07		
Training	0.01	0.03	0.01	0.20	0.12	0.07	

## Data Availability

The data presented in this study are available on request from the corresponding author. The data are not publicly available due to ethical.

## Notes

1 The percentage of disability is officially determined by a National Health Service evaluation and is officially recognized throughout the state. This means that the National Health Service Assessment and Guidance Teams apply a nationally established scale to identify the degree of disability a person may have.

2 Own studies in Spanish universities refer to the set of degrees recognized by the university itself. They are not approved or regulated at the state level. More information at: https://www.um.es/web/estudios-propios/.

3 To reference the citations extracted from the data, the coding offered by the ATLAS.ti 22 program has been used. The first number indicates the document where the appointment is located and the second is the appointment within said document.

4 Different coefficients of co-occurrence offered by the software will be shown, indicating the intensity or strength of relationship between two codes based on the number of times they appear simultaneously in a quotation. With respect to the strength of the relationship between two codes, it is not necessary to arrive at a minimum coefficient for consideration. It is necessary to analyze them individually.

5 In the different figures facilitated by the ATLAS.ti software, a greater intensity of relationship is reflected in proportionally darker colours, in addition to presenting a higher coefficient of co-occurrence, as indicated above. Yellow circles indicate that the coefficient is out of range, so it is not considered for use in the results.
